# Treatment diversification is an essential strategy for mitigating antimicrobial resistance

**DOI:** 10.1093/haschl/qxaf032

**Published:** 2025-03-10

**Authors:** Lao-Tzu Allan-Blitz, Jeffrey D Klausner

**Affiliations:** Division of Global Health Equity: Department of Medicine, Brigham and Women's Hospital, Boston, MA 02115, United States; Department of Population and Public Health Sciences, Keck School of Medicine, University of Southern California, Los Angeles, CA 90033, United States

Dear Editor,

We read with great interest the article by Spellberg et al,^1^ which advocated for sustainable solutions for antimicrobial resistance. We commend the authors for highlighting key strategies, including incentivizing antimicrobial stewardship, funding programs that decrease reliance on antibiotics, and a partnership between nonprofit and for-profit drug-development organizations. Those directions make sense; however, the authors neglected an essential strategy for mitigating the continued rise of antimicrobial-resistant infections: treatment diversification.

Antibiotic selection prior to susceptibility testing often results in empiric one-size-fits-all treatment for a given pathogen. *Neisseria gonorrhoeae,* 1 of the top 5 urgent antimicrobial-resistant threats to public health,^2^ is nearly exclusively treated with ceftriaxone. It is not surprising, therefore, that strains of *N gonorrhoeae* harboring high-level ceftriaxone resistance have now achieved sustained international transmission. Diversifying therapy at the point-of-care requires the availability of 1 of 2 options: effective alternative agents or rapid resistance tests.

For *N gonorrhoeae,* 2 new antimicrobials are in late-stage development and are expected to soon enter the market. Modeling work supports the need to implement the novel regimens rapidly and simultaneously to prolong the duration of effectiveness before resistance develops, rather than in sequence.^3^ A similar approach can be applied to numerous other infections with available alternative therapies; provider- and institution-level treatment practices per pathogen must attempt to use a variety of effective options. Antimicrobial stewardship has traditionally focused on minimizing the use of unnecessary antibiotics and of agents with unnecessarily broad coverage; however, diversifying the selection of empiric regimens will further reduce selective pressure towards resistance.

Rapid molecular assays that are commercially available predict resistance for a range of drug–pathogen combinations, including ciprofloxacin resistance in *N gonorrhoeae,* macrolide resistance in *Mycoplasma genitalium,* methicillin resistance in *Staphylococcus aureus,* and clarithromycin and levofloxacin resistance in *Helicobacter pylori*. The use of such assays has been incorporated into treatment guidelines, is increasingly common, and has been shown in prospective studies to be effective.^4^ Further, modeling work in *N gonorrhoeae* demonstrated that resistance-guided therapy can extend the usefulness of ceftriaxone by several years.^5^

Treatment diversification can and should be incentivized through the same mechanisms outlined by Spellberg et al. Increasing the variety of effective empiric regimens should be a topic within antimicrobial stewardship programs, while novel compound development will benefit from prioritizing the parallel development of resistance assays to facilitate appropriate usage. Treatment diversification must be an essential component of sustainable solutions for mitigating the continued emergence of antimicrobial resistance.

## Supplementary Material

qxaf032_Supplementary_Data
